# Supplementary material for: Deduplication Improves Cost-Efficiency and Yields of De Novo Assembly and Binning of Shotgun Metagenomes in Microbiome Research
Source: Microbiol Spectr. 2023 Feb 6;11(2):e04282-22. doi: 10.1128/spectrum.04282-22 (PMC10101064; doi:10.1128/spectrum.04282-22)
Supplement: Supplemental file 1 — Fig. S1 to S7. Download spectrum.04282-22-s0001.pdf, PDF file, 1.8 MB [file spectrum.04282-22-s0001.pdf]

SUPPLEMENTARY MATERIAL for

Deduplication Improves Cost-Efficiency and Yields of *De novo* Assembly and Binning of Shot-Gun Metagenomes in Microbiome Research

*** Corresponding to:**

Dr. Feng Ju (Assistant Professor)

Address: Environmental Microbiome and Biotechnology Laboratory (EMBLab)

Westlake University, Hangzhou 310030, China

Tel.: 571-87963205 (lab), 571-87380995 (office)

Fax: 0571-85271986

E-mail: jufeng@westlake.edu.cn

Supplementary figures

Fig. S1. The correlation between duplication rate and sequencing depth.

Fig. S2. The relationship between microbiome complexity and assembly quality.

Fig. S3. The number of MAGs recovered from MEGAHIT and metaSPAdes assemblies of standard and deduplicated data.

Fig. S4. The species-level MAG congruence recovered from standard data and deduplicated data.

Fig. S5. The disagreement rate of taxonomic annotations of 10 marker genes in MAGs recovered from standard data and deduplicated data.

Fig. S6. Time consumption and memory requirement of binning for the standard data and deduplicated data.

Fig. S7. The number of MAGs recovered from MEGAHIT and metaSPAdes assemblies by cross-sample binning and individual-sample binning strategies.

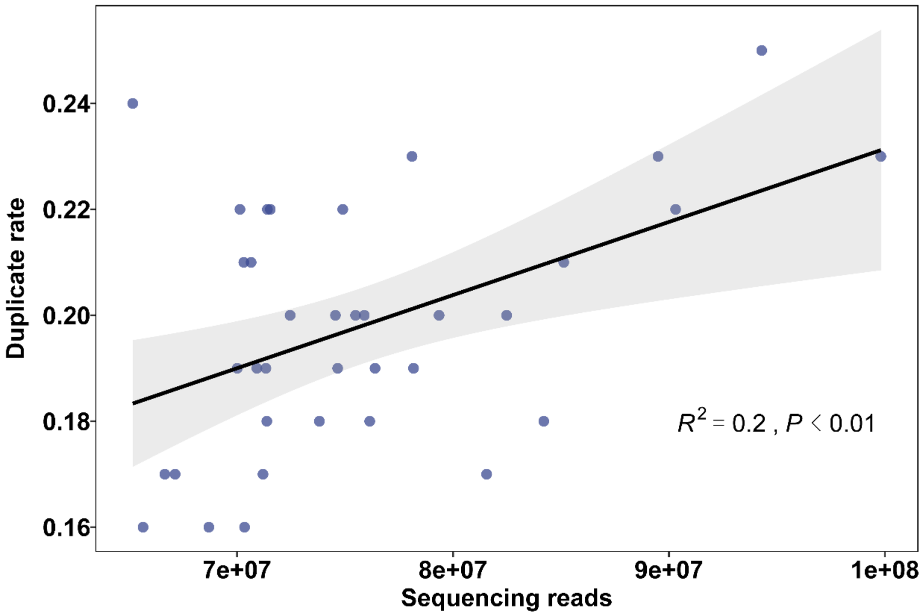

Fig. S1. The correlation between duplication rate and sequencing depth.

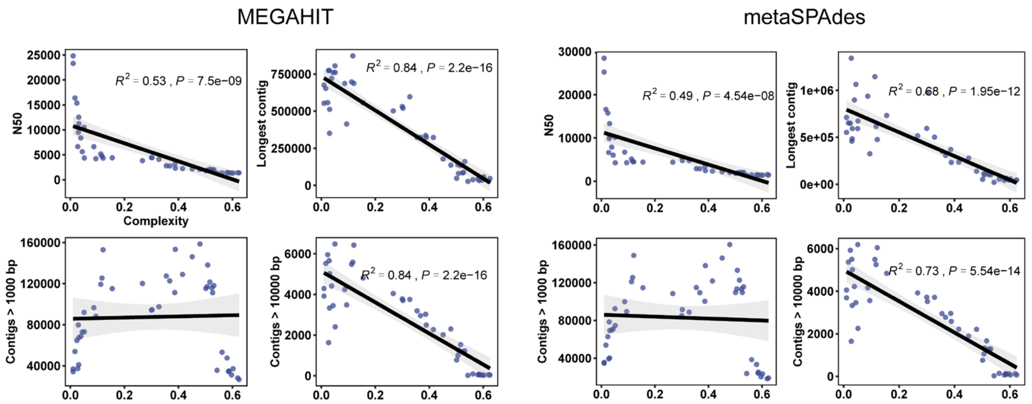

Fig. S2. The regression analysis of microbiome complexity and assembly quality of the 45 metagenomes studied. Microbiome complexity was evaluated by 1- estimated average coverage as shown in Fig. 1.

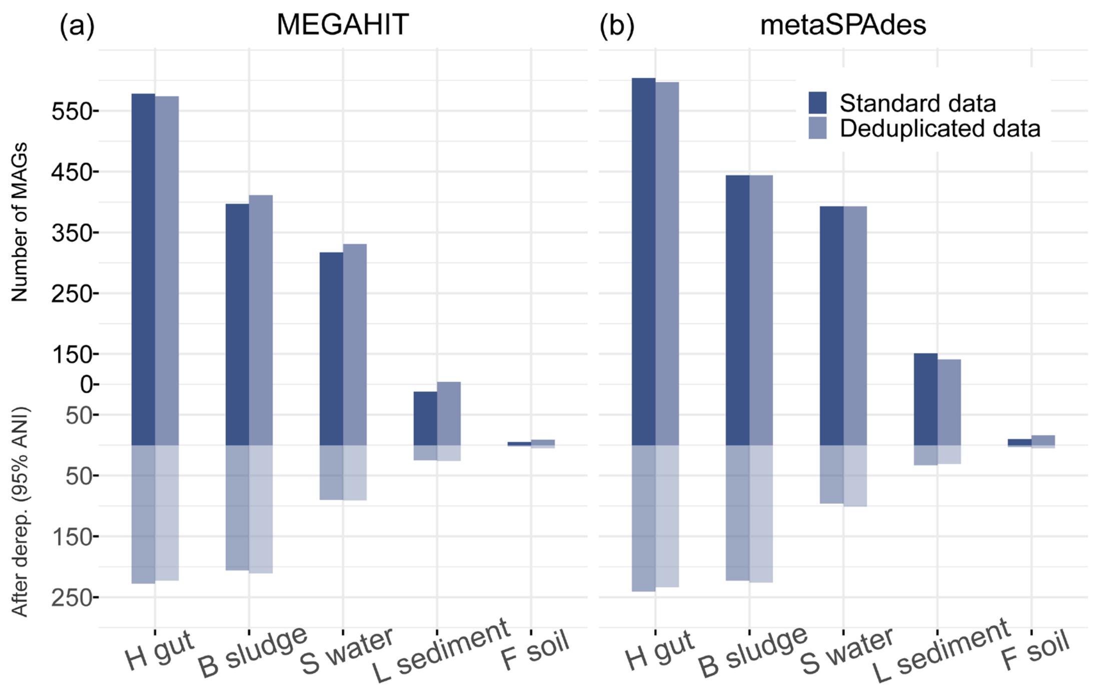

Fig. S3. The number of MAGs recovered from MEGAHIT and metaSPAdes assemblies of standard and deduplicated data. The dereplication was conducted with cutoff of ANI > 95%. H gut, B sludge, S water, L sediment and F soil indicate human gut, bioreactor sludge, surface water, lake sediment and forest soil metagenomes.

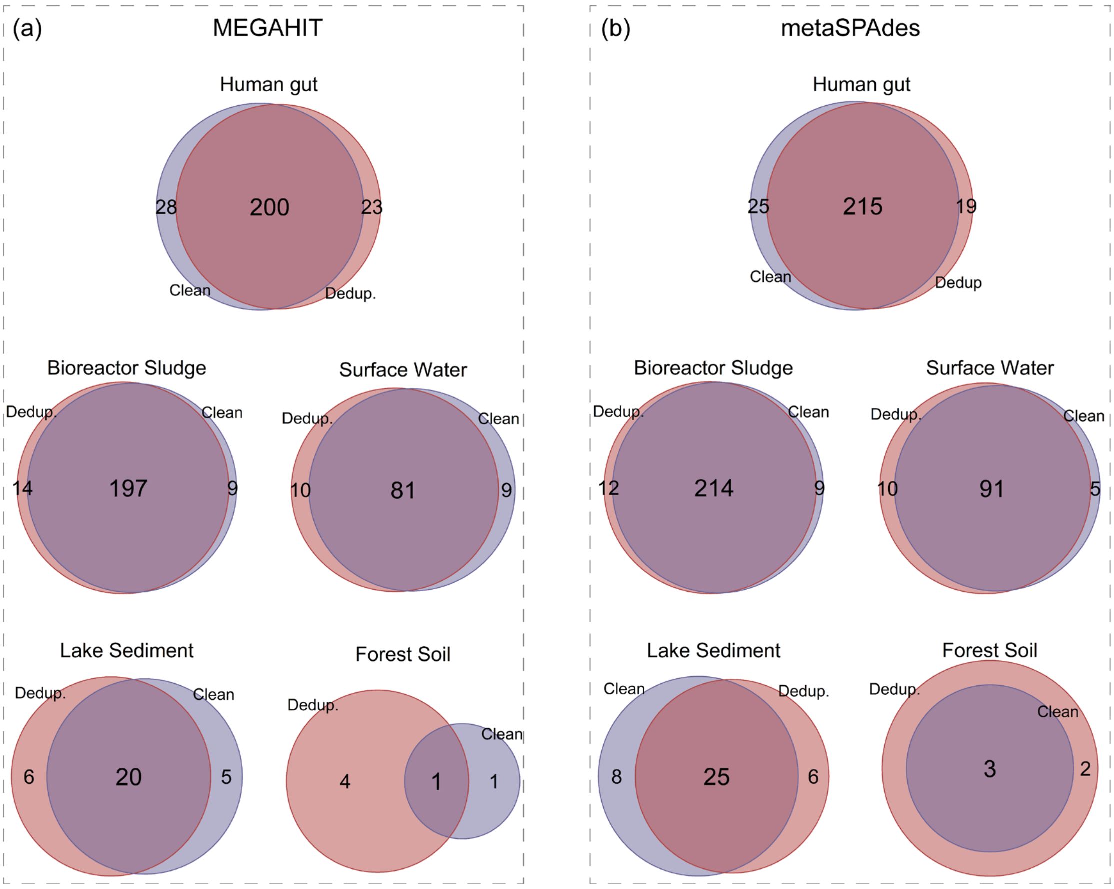

Fig. S4. The species-level MAG congruence recovered from deduplicated data and standard data. An MAG will be considered to be recovered from both standard data and deduplicated, if it has a counterpart with > 98% ANI.

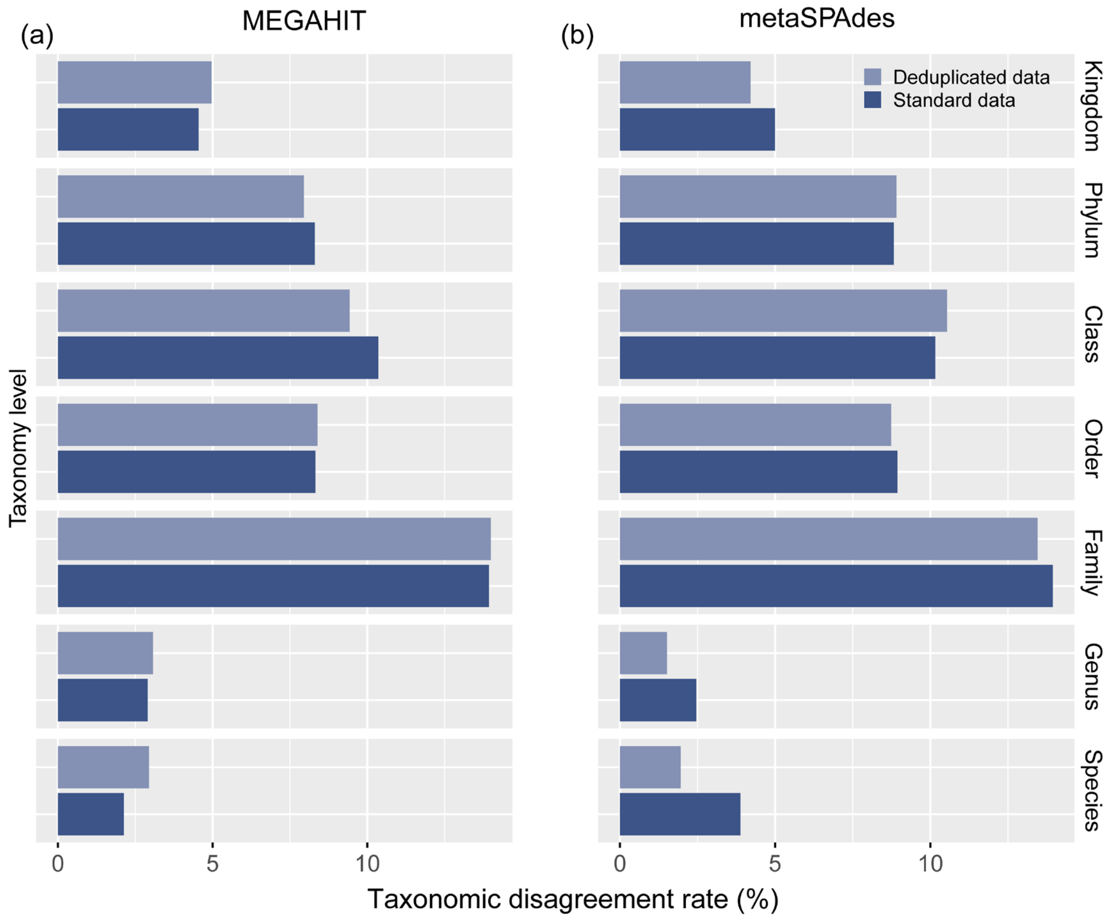

Fig. S5. The disagreement rate of taxonomic annotations of 10 marker genes in MAGs recovered from standard data and deduplicated data.

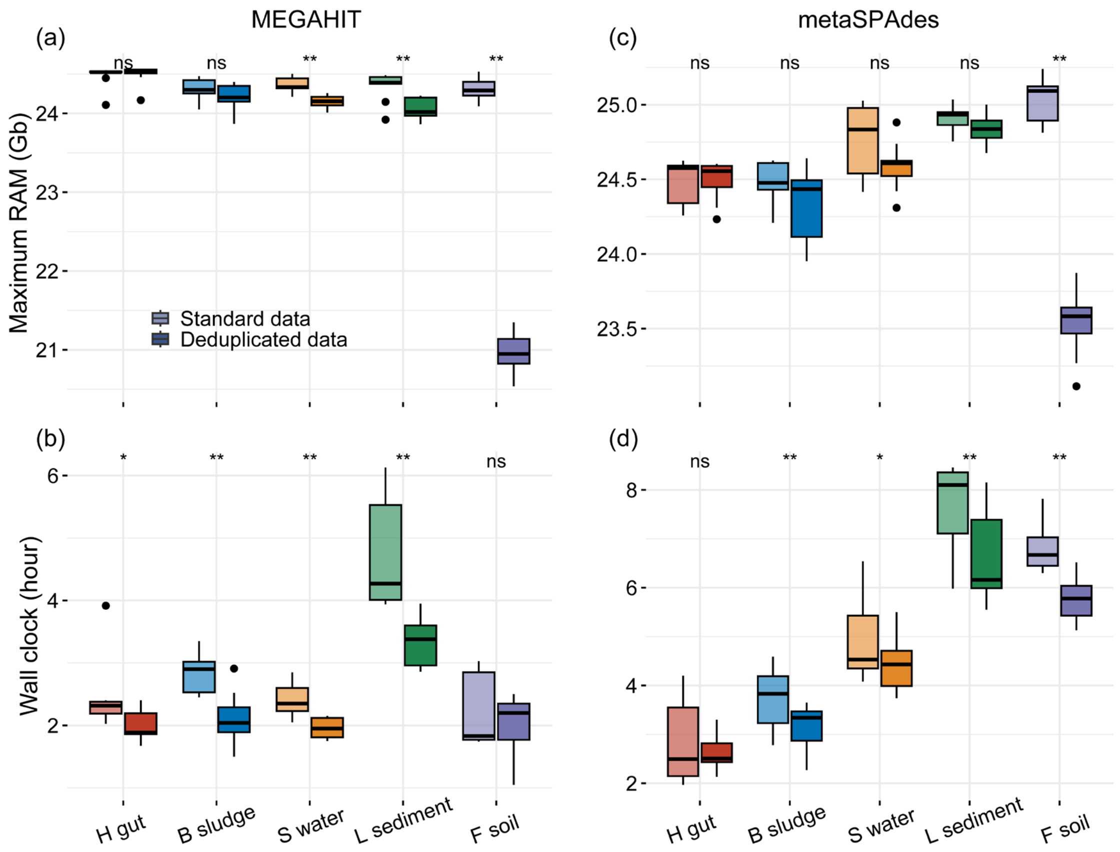

Fig. S6. Time consumption and memory requirement of binning for the standard data and deduplicated data. Significance was checked using Wilcoxon signed-rank test. “ns”, “*” and “**” indicate $p > 0.05$, $p < 0.05$ and $p < 0.01$, respectively.

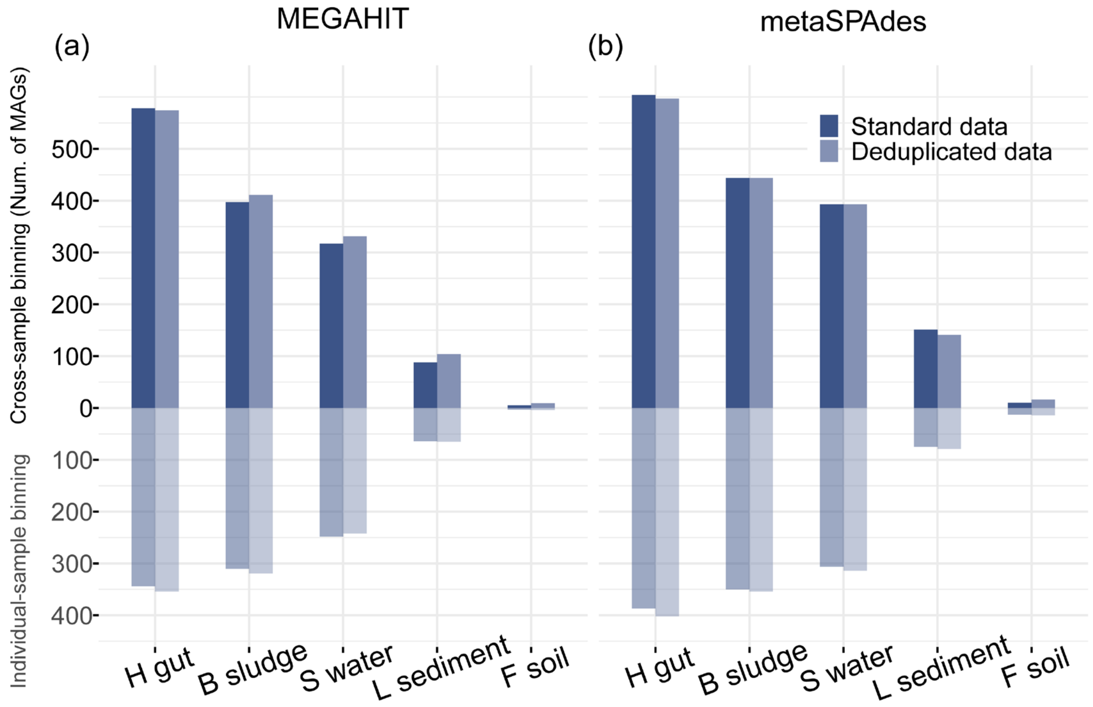

Fig. S7. The number of MAGs recovered from MEGAHIT and metaSPAdes assemblies by cross-sample binning and individual-sample binning strategies.
